# Demographics and clinical characteristics associated with sustained remission and continuation of sustained remission in patients with rheumatoid arthritis treated with adalimumab

**DOI:** 10.1186/s41232-019-0094-0

**Published:** 2019-03-25

**Authors:** Noriko Kimura, Katsuya Suzuki, Tsutomu Takeuchi

**Affiliations:** Division of Rheumatology, Department of Internal Medicine, Keio University School of Medicine, 35 Shinanomachi Shinjuku-ku, Tokyo, 160-8582 Japan

**Keywords:** Rheumatoid arthritis, Adalimumab, Sustained remission, Factors associated with sustained remission

## Abstract

**Background:**

Tumor necrosis factor inhibitor therapy for rheumatoid arthritis (RA) patients reduces disease activity, but little is known about the factors that correlate with continuation of remission. To identify demographics and clinical characteristics associated with achievement and continuation of sustained remission in patients with rheumatoid arthritis treated with adalimumab (ADA).

**Methods:**

In this retrospective cohort study, clinical outcome was retrospectively evaluated in RA patients that received ADA at a single institution using 28-joint disease activity score with erythrocyte sedimentation rate (DAS28-ESR). Sustained remission was defined as DAS28-ESR < 2.6 for more than 6 months, and continuation of sustained remission was defined as DAS28-ESR < 2.6 that was maintained until the end of the observation period.

**Results:**

Of 122 patients undergoing treatment with ADA between July 2008 and April 2014, 39 (32.0%) achieved sustained remission, and 22 of the 39 (56.4%) continued sustained remission until the end of the observation period (median, 20.5 months). Four of the 39 patients discontinued ADA because of remission, but 3 of these 4 patients restarted ADA because of RA flare. DAS28-ESR at the time of achievement of remission was lower in the subgroup of patients with continuation of sustained remission than the subgroup with RA flare.

**Conclusion:**

Of 122 patients, 39 (32.0%) achieved remission that was sustained for more than 6 months and 22 of the 39 patients (56.4%) continued sustained remission until the end of the observation period. Continuation of sustained remission was correlated with DAS28-ESR at the time of achievement of remission.

## Background

Tumor necrosis factor inhibitor therapy for rheumatoid arthritis (RA) patients reduces disease activity and improves function. Adalimumab (ADA) is a tumor necrosis factor inhibitor with well-established efficacy [1, 2]. Factors that predict RA remission with ADA treatment include concomitant use of methotrexate (MTX), no prior use of biologic disease-modifying antirheumatic drugs (DMARDs) [3], negative anti-citrullinated protein antibody (ACPA), low 28-joint disease activity score with erythrocyte sedimentation rate (DAS28-ESR), no erosion, and low disability index of the health assessment questionnaire (HAQ-DI) [4]. However, little is known about the factors that correlate with the continuation of remission. In addition, clinical response to ADA is dependent on ethnicity; therefore, the clinical course of Japanese patients after remission may be very different to those reported for other populations [5].

We attempted to identify factors associating with sustained remission and continuation of sustained remission in RA patients treated with ADA.

## Methods

### Study design and patients

This study was a single center, retrospective cohort study. RA patients who started ADA treatment at our institution between July 2008 and April 2014 were enrolled. All patients satisfied the classification criteria of the American College of Rheumatology [6]. Patient information was obtained by retrospective analysis of medical records.

### Clinical efficacy

Disease activity was assessed using DAS28-ESR [7]. We defined sustained remission as a DAS28-ESR< 2.6 that was maintained for more than 6 months, and compared the background characteristics of those who did and did not achieve sustained remission. We separated the sustained remission group into two subgroups: patients who maintained remission until the end of the observation period (defined as continuation of sustained remission) and those who experienced RA flare. Differences between these two subgroups were evaluated. The end of the observation period was defined as the period up until ADA was discontinued or the last observation (November 2014). Functional disability was assessed using HAQ-DI [8].

### Statistical analysis

The baseline characteristics of patients were summarized as mean, standard deviation (SD), and median. Demographic and baseline characteristics were analyzed using the Wilcoxon rank-sum test for continuous variables and the chi-square test for categorical variables. Significant variables in univariate models were included in multivariate models to identify factors that correlated with sustained remission rate. The presence of a statistically significant difference in disease activity was analyzed using the one-way analysis of variance. JMP® (SAS Institute Inc.) was used for statistical analysis.

## Results

### Baseline characteristics

A total of 122 RA patients were administered ADA between July 2008 and April 2014. Of the 122 patients, 7 received 80 mg of ADA every other week during therapy and 115 received 40 mg of ADA. Baseline demographics and disease characteristics are summarized in Table 1. Mean age ± SD of the 122 patients was 56.9 ± 16.3 years and the majority were women (81.1%). Mean body weight was 53.8 ± 10.5 kg and mean disease duration was 8.2 ± 9.4 years. Distribution of disease activity (DAS28-ESR) was as follows: high (5.1 <): 30 cases, moderate (3.2 < DAS ≤ 5.1): 68 cases, low (≤ 3.2): 12 cases, and not applicable: 12 cases. Mean baseline DAS28-ESR score was 4.48 ± 1.24 and HAQ-DI score was 0.95 ± 0.80. In total, 101 (82.8%) patients received concomitant MTX at a mean dose of 9.8 ± 3.1 mg/week and 36 (29.5%) received concomitant prednisolone at a mean dose of 5.0 ± 2.8 mg/day at the beginning of ADA treatment. Of the 122 patients, 87 (71.3%) were naïve to biological DMARD treatment, while 35 (28.7%) had received biological DMARDs prior to ADA. Of the latter 35 patients, 10 had received more than 2 biological DMARDs, and 21, 19, 7, and 5 patients had received infliximab, etanercept, tocilizumab, and ADA, respectively. Among five patients, in two patients ADA was used as a clinical trial before this study period. After ADA was stopped, then ADA was restarted in the observation period of this study. In another three patients, ADA was used two times in different period in this study.

**Table 1:** Baseline characteristics of patients

Variable	Total (*n* = 122)
Female, *n* (%)	99 (81.1)
Age, years	56.9 ± 16.3 (59.0)
Weight, kg	53.8 ± 10.5 (52.0)
Disease duration, years	8.2 ± 9.4 (4.0)
DAS28-ESR	4.48 ± 1.24 (4.29)
Tender joint count	3.6 ± 4.7 (2)
Swollen joint count	5.2 ± 4.3 (4)
Physician’s VAS	35.5 ± 21.3 (30)
Patient’s VAS	40.2 ± 25.3 (36.5)
ESR, mm/h	47.2 ± 35.6 (41)
CRP, mg/dL	1.55 ± 2.14 (0.53)
RF-positive, *n* (%)	84 (68.9)
ACPA-positive, *n* (%)	77 (73.3)
Stage I/II/III/IV, %	25.4/37.3/2.5/34.7
HAQ-DI	0.95 ± 0.80 (0.75)
MTX use, *n* (%)	101 (82.8)
MTX dose, mg/week	9.8 ± 3.1 (10.0)
Oral steroid use, *n* (%)	36 (29.5)
Oral steroid dose, mg/day	5.0 ± 2.8 (5.0)
Prior use of biologics, *n* (%)	35 (28.7)

Data are mean ± SD (median) unless otherwise indicated

*DAS* disease activity score, *ESR* erythrocyte sedimentation rate, *RF* rheumatoid factor, *ACPA* anti-citrullinated protein antibody, *HAQ-DI* Health Assessment Questionnaire Disability Index, *MTX* methotrexate

### Evaluation of baseline factors associating with sustained remission

The proportion of patients who received concomitant MTX treatment was significantly higher in the patient group that achieved sustained remission compared to the group that did not. In contrast, age, disease duration, DAS28-ESR and HAQ-DI at baseline, the proportion of patients on concomitant prednisolone, and the proportion of patients with a history of prior use of biologics were higher in the patient group that did not achieve sustained remission compared to the group that did (Table 2). Multivariate analysis showed that prior use of biologics was significantly associated with the achievement of sustained remission (Table 3).

**Table 2:** Baseline characteristics correlated with sustained remission

Variable	Sustained remission (*n* = 39)	No sustained remission (*n* = 83)	*P* value
Female, *n* (%)	34 (87.2)	65 (78.3)	0.24
Age, years	50.3 ± 18.8 (53.0)	60.1 ± 14.0 (62.0)	0.0067
Weight, kg	52.9 ± 9.1 (51.5)	54.2 ± 11.2 (53.0)	0.70
Disease duration, years	4.9 ± 7.2 (1.8)	9.8 ± 10.0 (7.0)	0.0061
DAS28-ESR at baseline	4.12 ± 0.88 (4.09)	4.68 ± 1.37 (4.48)	0.014
RF-positive, *n* (%)	25 (64.1)	59 (71.1)	0.44
ACPA-positive, *n* (%)	26 (72.2)	51 (73.9)	0.85
Presence of bone erosion, *n* (%)	26 (68.4)	63 (77.8)	0.27
HAQ-DI at baseline	0.63 ± 0.69 (0.38)	1.16 ± 0.82 (1.00)	0.0009
MTX use, *n* (%)	38 (97.4)	63 (75.9)	0.0033
Oral steroid use, *n* (%)	6 (15.4)	30 (36.1)	0.019
Prior use of biologics, *n* (%)	3 (7.7)	32 (38.6)	0.0004

Data are mean ± SD (median) unless otherwise indicated

*DAS* disease activity score, *ESR* erythrocyte sedimentation rate, *RF* rheumatoid factor, *ACPA* anti-citrullinated protein antibody, *HAQ-DI* Health Assessment Questionnaire Disability Index, *MTX* methotrexate

**Table 3:** Multivariate logistic regression analysis of baseline characteristics associated with sustained remission

Variable	χ2	*P* value
Age, years	3.0	0.84
Disease duration, years	0.79	0.37
DAS28-ESR at baseline	1.01	0.31
HAQ-DI at baseline	1.48	0.22
MTX dose, mg weekly	0.26	0.61
PSL dose, mg daily	0.73	0.39
Prior use of biologics	5.7	0.017

*DAS* disease activity score, *ESR* erythrocyte sedimentation rate, *HAQ-DI* Health Assessment Questionnaire Disability Index, *MTX* methotrexate

### Evaluation of factors that correlated with the continuation of sustained remission

**Fig. 1:**
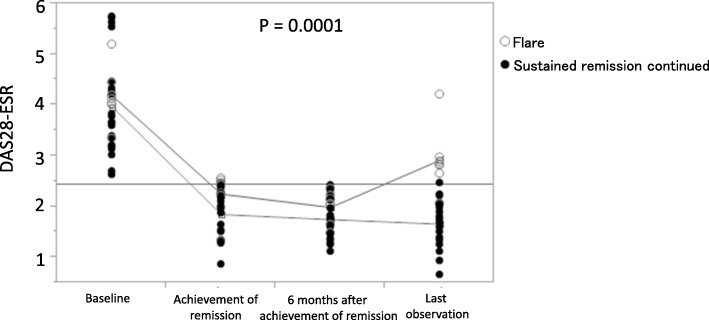
The clinical course of patients who continued sustained remission and those who experienced RA flare. At all three points, disease activity were significantly reduced from baseline. (*P* value < 0.0001, one-way ANOVA test)

Of the 39 patients who achieved sustained remission, 4 discontinued ADA because of sustained remission, 22 continued sustained remission and treated with ADA until the end of the observation period (mean ± SD, 27.8 ± 18.6 months; median, 20.5 months), 7 experienced RA flare (2 switched from ADA to other biologic DMARDs and 5 continued ADA), and 6 discontinued ADA because of reasons other than remission (2 because of pregnancy, 2 because of a change of hospital, 1 because of cerebral infarction, and 1 because of patient request). The patient subgroup that experienced RA flare had higher DAS28-ESR at the time of achievement of remission compared to the group that continued sustained remission (Table 4). Similarly, the subgroup of patients who experienced RA flare had higher DAS28-ESR throughout the observation period compared with the subgroup that continued sustained remission (Fig. 1). Regarding MTX dose, dose at achievement of remission showed no significant change between two groups (Table 4). In the seven patients of flare, mean MTX dose at flare up was 7 mg/week (range 0–15). In two patients, MTX was stopped due to side effects. (One was due to lymphoproliferative disorder, and another was due to liver dysfunction and aphthous ulcer.) Among another five patients, in one case, mean MTX dose at flare up was markedly reduced from 16 to 4 mg/week. In another four cases, MTX dose at flare up was not markedly reduced. This observation may indicate that reduction of MTX dose after remission becomes a potential risk factor of flare up. Regarding corticosteroid, in the seven patients of flare, in only a patient, dose reduction of corticosteroid (5 to 0 mg/day as predonisolone) was observed. Another one patient had no change (2 mg/day as predonisolone). The remaining five patients did not use corticosteroid. Elongation of treatment interval of ADA was not confirmed in the seven patients.

**Table 4:** Factors associated with the continuation of sustained remission

Variable	Sustained remission continued (*n* = 22)	Flare (*n* = 7)	*P* value
Female, *n* (%)	18 (81.9)	6 (85.7)	0.81
Age, years	49.8 ± 14.2 (52)	54.4 ± 23.9 (63)	0.33
Weight, kg	55.0 ± 9.5 (53)	47.9 ± 8.3 (46)	0.99
Disease duration, years	3.6 ± 4.5 (1.8)	9.2 ± 11.3 (2)	0.15
Time until achievement of remission	3.8 ± 3.3 (3)	2.0 ± 1.9 (1)	0.13
DAS28-ESR at baseline	3.97 ± 0.96 (3.73)	4.19 ± 0.56 (4.13)	0.41
DAS28-ESR at achievement of remission	1.85 ± 0.44 (1.96)	2.25 ± 0.43 (2.42)	0.027
RF-positive, *n* (%)	12 (54.6)	4 (57.1)	0.90
ACPA-positive, *n* (%)	13 (61.9)	4 (66.7)	0.83
Presence of bone erosion, *n* (%)	12 (57.1)	5 (71.4)	0.50
HAQ-DI at baseline	0.18 ± 0.36 (0)	0.52 ± 1.11 (0)	0.69
HAQ-DI at achievement of remission	0.23 ± 0.40 (0)	0.54 ± 1.06 (0)	0.76
MTX at baseline, mg/week	10.8 ± 3.01 (10)	12.5 ± 3.45 (12.5)	0.21
MTX at achievement of remission, mg/week	10.6 ± 2.79 (10)	12.0 ± 3.00 (12)	0.26
Oral steroid use, *n* (%)	3 (13.6)	2 (28.6)	0.36
Prior use of biologics, *n* (%)	3 (13.6)	0 (0)	0.30
Observation period, months	27.8 ± 18.6 (20.5)	32.4 ± 21.4 (23.0)	0.54

Data are mean ± SD (median) unless otherwise indicated

*DAS* disease activity score, *ESR* erythrocyte sedimentation rate, *RF* rheumatoid factor, *ACPA* anti-citrullinated protein antibody, *HAQ-DI* Health Assessment Questionnaire Disability Index, *MTX* methotrexate

### Course of four patients who discontinued ADA because of sustained remission

The characteristics of four patients who discontinued ADA because of sustained remission are summarized in Table 5. Three of the four patients experienced RA flare after ADA discontinuation.

**Table 5:** Characteristics of four patients who discontinued ADA because of sustained remission

Variable	Case 1	Case 2	Case 3	Case 4
Sex	Female	Female	Female	Female
Age, years	38	63	69	80
Weight, kg	54	42	53	62
Disease duration, years	4	0.3	3.2	0.8
DAS28-ESR at baseline	4.34	5.92	4.09	4.96
RF	58	< 5	35	80
ACPA	> 100	< 0.6	59.2	> 300
Stage	II	I	IV	II
HAQ-DI at baseline	No data	1.625	1.125	0.75
MTX mg/week	8	6	8	6
Prior use of biologics	No	No	No	No
Time until achievement of remission, months	1	12	1	1
Continuation of remission, months	29	12	12	34
DAS28-ESR at discontinuation of ADA	0.97	2.11	1.46	2.50
HAQ-DI at discontinuation of ADA	No data	0.75	0	0
First outcome	Flare at 6 months	Remission continued for 29 months	Flare at 8 months	Flare at 3 months
Second outcome	Achieved remission 1 month after restarting ADA	No flare	No remission after restarting ADA	Achieved remission 1 month after restarting ADA

*DAS* disease activity score, *ESR* erythrocyte sedimentation rate, *RF* rheumatoid factor, *ACPA* anti-citrullinated protein antibody, *HAQ-DI* Health Assessment Questionnaire Disability Index, *MTX* methotrexate

## Discussion

We retrospectively identified factors that correlated with sustained remission and continuation of sustained remission. We found that sustained remission was associated with age, disease duration, DAS28-ESR and HAQ-DI at baseline, concomitant MTX, no concomitant prednisolone use, and no prior use of biologics. Continuation of sustained remission was associated with DAS28-ESR at the time of achievement of remission. A small number of RA patients discontinued ADA because of remission, with some experiencing a subsequent flare up.

Patients of the HONOR study [9], an open-label, non-randomized study of Japanese patients with established RA and DAS28-ESR < 2.6 for 6 months while under ADA treatment, had shorter disease duration, lower scores for patient global assessment and HAQ-DI, and lower ESR and DAS28-ESR. Further, the proportion of patients with DAS28-ESR < 2.6 for 1 year was significantly lower (48%) in the ADA discontinuation group than in the ADA continuation group (83%) after remission was maintained for 6 months. Furthermore, reinitiating ADA resulted in the reinduction of low disease activity (LDA) by 90% within 6 months and 100% within 9 months without any harmful effects.

Our results concerning disease activity were similar to those of the HONOR study. Future studies should evaluate structural and functional remission. The HOPEFUL 2 study, a postmarketing observational study of early RA patients, demonstrated that 93% of patients among those with DAS28-CRP ≤ 2.0 at ADA discontinuation achieved sustained LDA for 1 year. This suggested that DAS28 remission might predict biologic-free disease control in patients with early RA [10]. Further, 80% (12/15) of patients who discontinued ADA after achieving stable remission with ADA and MTX treatment had a flare up in the 6 months after discontinuation [11].

In our study, 56% (22/39) of patients who achieved sustained remission for more than 6 months maintained sustained remission until the end of the observation period (mean ± SD, 27.8 ± 18.6 months; median, 20.5 months), and lower DAS28-ESR at the time of achievement of remission correlated with continuation of sustained remission. Among the patients who discontinued ADA after achieving sustained remission, 75% (3/4) experienced a flare up, and two of three patients achieved remission again 1 month after reinitiating ADA. Therefore, discontinuation of ADA after achieving remission should be decided with caution, with consideration given to the background of the patients.

In short-term sustained remission, no biologics history was a significant favorable factor (Tables 2 and 3). On the other hand, in long-term sustained remission, the number of prior use of biologics showed no significant difference between sustained remission continued and flare groups. In sustained remission continued group (*n* = 22), three cases had history of prior use of biologics (infliximab: 1, infliximab and etanercept: 2), whereas no history in flare group (*n* = 7). This discrepancy may be caused from the following possibilities. In later analysis, proportion of prior use of biologics was lower (3/29 vs 35/121) and statistical power may be insufficient. Furthermore, in sustained remission continued group may not be homogeneous. To clarify the difference of mode of action in biologics, analysis in further larger cohort is needed.

In HONOR study [9], DAS28-ESR ≤ 1.98 was defined as “deep remission.” In our cohort, mean and median DAS28-ESR at achievement of remission in sustained remission continued group were 1.85 and 1.96, whereas 2.25, 2.42 in flare group (Table 4). Cut off value “1.98” of deep remission in HONOR study is comparable to our results. On the other hand, in four patients who discontinued ADA because of sustained remission, two patients with low DAS28-ESR (0.97 and 1.46) at achievement of remission flared at 6 and 8 months (Table 5). We thought DAS28-ESR at achievement of remission was an important indicator for long-term sustained remission, but still unclear in discontinued ADA because of sustained remission.

Finally, we should note study limitation. Some of the patients reduced the dose of MTX or corticosteroid or may have elongated the treatment interval of ADA. Such treatment change might have affected the flare of disease.

## Conclusion

Of 122 patients, 39 (32.0%) achieved remission that was sustained for more than 6 months and 22 of the 39 patients (56.4%) continued sustained remission until the end of the observation period. Continuation of sustained remission was correlated with DAS28-ESR at the time of achievement of remission.

## Abbreviations

ADA: Adalimumab
DAS28-ESR: 28-Joint disease activity score with erythrocyte sedimentation rate
DMARDs: Disease-modifying antirheumatic drugs (DMARDs)
HAQ-DI: Health assessment questionnaire (HAQ-DI)
MTX: Methotrexate
RA: Rheumatoid arthritis
SD: Standard deviation

## References

[1] Breedveld FC, Weisman MH, Kavanaugh AF (2006). The PREMIER study: a multicenter, randomized, double-blind clinical trial of combination therapy with adalimumab plus methotrexate versus methotrexate alone or adalimumab alone in patients with early, aggressive rheumatoid arthritis who had not had previous methotrexate treatment. Arthritis Rheum.

[2] Smolen JS, Emery P, Fleischmann R (2014). Adjustment of therapy in rheumatoid arthritis on the basis of achievement of stable low disease activity with adalimumab plus methotrexate or methotrexate alone: the randomised controlled OPTIMA trial. Lancet.

[3] Takeuchi T, Tanaka Y, Kaneko Y (2012). Effectiveness and safety of adalimumab in Japanese patients with rheumatoid arthritis: retrospective analyses of data collected during the first year of adalimumab treatment in routine clinical practice (HARMONY study). Mod Rheumatol.

[4] Takeuchi T, Yamanaka H, Ishiguro N (2014). Adalimumab, a human anti-TNF monoclonal antibody, outcome study for the prevention of joint damage in Japanese patients with early rheumatoid arthritis: the HOPEFUL 1 study. Ann Rheum Dis.

[5] Takeuchi T, Kameda H (2010). The Japanese experience with biologic therapies for rheumatoid arthritis. Nat Rev Rheumatol.

[6] Arnett FC, Edworthy SM, Bloch DA (1988). The American rheumatism association 1987 revised criteria for the classification of rheumatoid arthritis. Arthritis Rheum.

[7] Prevoo MLL, van’t Hof MA, Kuper HH (1995). Modified disease activity scores that include twenty- eight-joint counts. Arthritis Rheum.

[8] Fries JF, Spitz P, Kraines RG (1980). Measurement of patient outcome in arthritis. Arthritis Rheum.

[9] Tanaka Y, Hirata S, Kubo S (2015). Discontinuation of adalimumab after achieving remission in patients with established rheumatoid arthritis: 1-year outcome of the HONOR study. Ann Rheum Dis.

[10] Tanaka Y, Yamanaka H, Ishiguro N (2016). Adalimumab discontinuation in patients with early rheumatoid arthritis who were initially treated with methotrexate alone or in combination with adalimumab: 1 year outcomes of the HOPEFUL-2 study. RMD open.

[11] Chatzidionysiou K, Turesson C, Teleman A (2016). A multicentre, randomised, controlled, open-label pilot study on the feasibility of discontinuation of adalimumab in established patients with rheumatoid arthritis in stable clinical remission. RMD Open.

